# IFNβ-Induced *CXCL10* Chemokine Expression Is Regulated by Pellino3 Ligase in Monocytes and Macrophages

**DOI:** 10.3390/ijms232314915

**Published:** 2022-11-29

**Authors:** Edyta Makuch, Izabella Jasyk, Anna Kula, Tomasz Lipiński, Jakub Siednienko

**Affiliations:** 1 Łukasiewicz Research Network—PORT Polish Center for Technology Development, 54-066 Wrocław, Poland; 2 Institute of Immunology and Experimental Therapy, Polish Academy of Sciences, 53-114 Wrocław, Poland

**Keywords:** IFNβ, CXCL10, Pellino3 ligase, monocytes, macrophages

## Abstract

IFN-I is the key regulatory component activating and modulating the response of innate and adaptive immune system to bacterial as well as viral pathogens. IFN-I promotes the expression of IFN-induced genes (ISG) and, consequently, the production of chemokines, e.g., CXCL10. Those chemokines control migration and localization of immune cells in tissues, and, thus, are critical to the function of the innate immune system during infection. Consequently, the regulation of IFN-I signaling is essential for the proper induction of an immune response. Our previous study has shown that E3 ubiquitin ligase Pellino3 positively regulates IFNβ expression and secretion. Herein, we examined the role of Pellino3 ligase in regulating *CXCL10* expression in response to IFNβ stimulation. Our experiments were carried out on murine macrophage cell line (BMDM) and human monocytes cell line (THP-1) using IFNβ as a IFNAR ligand. We demonstrate that Pellino3 is important for IFNβ-induced phosphorylation and nuclear translocation of STAT1/STAT2/IRF9 complex which interacts with *CXCL10* promoter and enhances its expression. In this study, we characterize a novel molecular mechanism allowing Pellino3-dependent modulation of the IFNβ-induced response in BMDM and THP-1 cell lines.

## 1. Introduction

Type I interferons (IFN-I) are pleiotropic cytokines produced in response to viral and bacterial infections. That abundant family of human and mouse interferons encompasses multiple IFNα subtypes: IFNβ, IFNε, IFNκ, IFNω, and IFNζ [1]. All type I interferons bind to common transmembrane receptors—IFNARs (interferon α/β receptors), heterodimer composed of two subunits, IFNAR1 and IFNAR2, which may associate with the Janus-activated kinases (JAKs). The JAK family in composed of four members: JAK1, JAK2, JAK3, and tyrosine kinase 2 (TYK2). The association of the two IFNAR subunits induced by IFNs allows JAK1 and TYK2 to form a functional signaling unit that promotes STATs (signal transducer and activator of transcription) or NF-κB (nuclear factor-kappa B) signaling pathways [2,3,4]. In one of the paths, activation of JAKs results in tyrosine phosphorylation of STAT1 and STAT2; which leads to their dimerization via Src-homology 2 (SH2)-domain-phosphotyrosine. Next, STAT1/STAT2 heterodimers associate with IRF9 and form transcriptionally active IFN-stimulated gene factor 3 (ISGF3). These complexes translocate into the nucleus and bind to ISRE sites in promoters of ISGs (increased IFN-induced genes) [5]. IFN-I can also induce NF-κB pathways by two parallel mechanisms. In both cases, activation of Tyk2, but not JAK1, is required for signal transduction [6]. In the canonical pathway, IFN-I induces STAT3, PI3K, and Akt binding to the Tyk2, which promotes IκBα degradation and NF-κB p50/p65 activation [3,6]. The noncanonical pathway is dependent on NIK and TRAF proteins which induce the processing of the p100/NF-κB2 precursor into p52 [4]. The IFN-activated NF-κB pathways balance the ability of IFN to induce antiviral response and apoptosis of infected cells but also promote cell survival by regulating the expression of specific ISGs, e.g., CXCL11 [3,7].

Interferon beta (IFNβ) is produced in rapid response to viral infection by innate immune cells, including macrophages and monocytes, as well as non-immune cells, such as fibroblasts and epithelial cells [8]. IFNβ induces a variety of effects, including anti-inflammatory and pro-inflammatory responses, and also regulates the secretion of chemokines driving the development and activation of all innate and adaptive immune effector cells [9]. In addition, IFNβ stimulation disrupts viral replication and slows down the growth of infected cells, making them more susceptible to apoptosis [10]. Furthermore, it has been shown that IFNβ modulates TNFα and IL-10 expression in peripheral blood mononuclear cells [11] and monocytes [12], as well as regulating chemokine expression, e.g., CXCL10 in macrophages [13].

The chemokine system is critical for the function of immune cells. It organizes the migration and localization of immune cells in lymph organs and other tissues by the exertion of chemotactic effects. It has been shown that CXCL10 and CXCL11 play a key role in inflammation during Hepatitis C Virus (HCV) infection. It was confirmed that either HCV recognition or poly:IC stimulation induces the expression of these chemokines. Additionally, IFNβ stimulation results in a significant increase of CXCL10 production [13,14]. Moreover, IFNγ shows potent synergy with TNFα in promoting the expression of CXCL10 and CXCL11 in vitro [15]. CXCL10 is produced by several cell types in different tissue and exhibits pleiotropic effects on a wide range of biological processes, including immunity, angiogenesis, and tumor metastasis. The involvement of CXCL10 in such important processes makes it a promising therapeutic target for various diseases. Still, its transcriptional regulation, secretion, and mechanism of action are not fully characterized. CXCL10 was initially identified in human U937 monocytic-cells (a histiocytic lymphoma cell line with monocytic characterization and origin) in the human placenta and spleen as a product of IFNγ induction [16]. Like other members of the chemokine subfamily, CXCL10 is a low molecular weight (10 kDa) protein that has been functionally described as a pro-inflammatory chemokine. Its main biological function involves the recruitment of monocytes, macrophages, and T cells to sites of inflammation [17]. *CXCL10* expression is driven by a promoter spanning the region 875 nucleotides upstream from the transcriptional start site. The promotor region of *CXCL10* contains several important regulatory elements, such as: sites for nuclear factor-κB (NF-κB), site for activator protein 1 (AP-1), site for CCAAT/enhancer-binding protein β (C/EBP-β), site for interferon-stimulated response element (ISRE), and IFN-γ-activated site factors (GAS) [18,19]. Depending on the stimulating factor, various regulatory elements in the *CXCL10* promoter are utilized. HCV infection and TLR3 and RIG-I ligands have been shown to promote *CXCL10* expression by ISRE and NF-κB binding sites. On the other hand, the binding site for AP-1 and C/EBP-β negatively regulates *CXCL10* expression during HCV infection [19]. In turn, IFNβ stimulation induces *CXCL10* expression through interferon regulatory factor (IRF)-1 and IRF-2 binding to ISRE within the *CXCL10* promoter [13].

It has been shown that E3 ubiquitin ligase Pellino3 regulates the secretion of type I IFN during the innate immune response. In the signaling pathway activated by TLR3, Pellino3 interacts with TRAF6, thereby inhibiting the induction of IRF7 and, consequently, the expression of IFNβ [20]. Similarly, Pellino3 negatively regulates signaling pathways activated by the TLR4 receptor. The oxidized form of low-density lipoproteins induces Pellino3- and IRAK1/4-dependent monoubiquitination of TANK protein, resulting in lower IFNβ expression in response to LPS [21]. Moreover, our recent study demonstrated that Pellino3 regulates VSV-induced CXCL10 production [22]. However, the effect of Pellino3 on INFβ-activated signaling has not yet been described.

Here we show for the first time that the ubiquitin ligase Pellino3 is required for IFNβ-induced expression of *CXCL10* via the Tyk2 kinase regulation pathway. Moreover, we show that IFNβ promotes two differently regulated signaling pathways leading to CXCL10 production. First pathway depends on Pellino3-independent activation of *CXCL10* expression via the NF-κB pathway. The second pathway is Pellino3-dependent and involves IFNβ-induced formation of STAT1/STAT2/IRF9 complex, followed by its nuclear translocation and recruitment to the *CXCL10* promoter, leading to transcriptional activation.

## 2. Results

### 2.1. Pellino3 Positively Regulates CXCL10 Induction via Tyk2 after IFNβ Treatment

**Figure 1:**
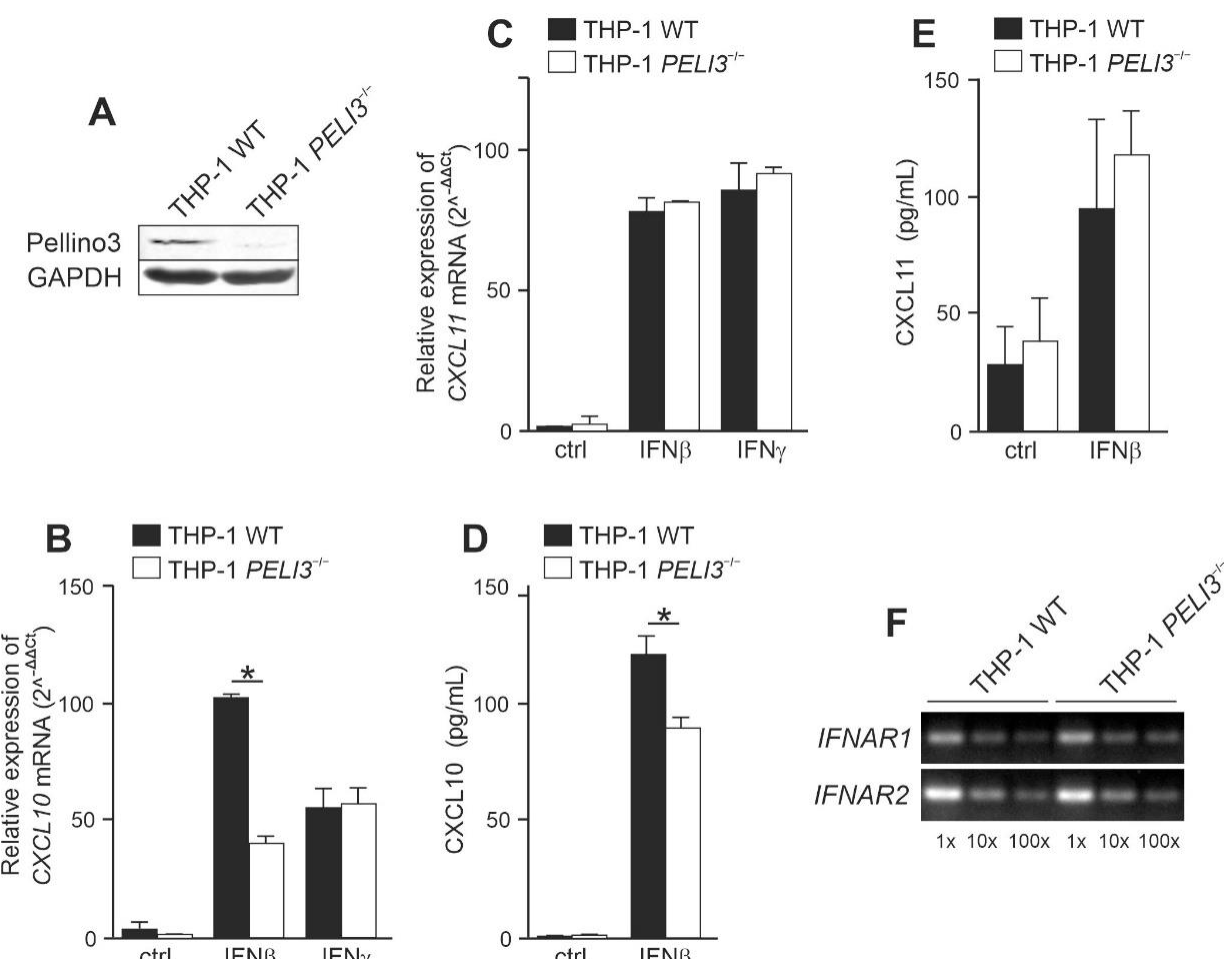
INFβ-dependent CXCL10 induction is downregulated in Pellino3 deficient THP-1 cell line. (**A**) Pellino3 knockout in THP-1 cells was generated using Guide-it CRISPR/Cas9 Gesicle Production System (Takara Bio) according to the manufacturer’s instruction. Cell lysates from WT and *PELI3*^−/−^ THP-1 were subjected to SDS-PAGE followed by Western blotting. Protein detection was performed using specific antibodies and appropriate secondary antibodies conjugated to the fluorescent dye in the infrared range. Visualization was performed using the Odyssey CLx Imaging System LI-COR. Wild type (WT) and *PELI3*^−/−^ THP-1 cells were treated with IFNβ (1000 U/mL) or IFNγ (15 ng/mL) for 4 h (**B**,**C**) or 16 h (**D**,**E**). (**B**,**C**) Thereafter, total RNA was isolated and reverse transcribed. Quantitative real-time PCR (2^–(ΔΔCT)^) was used to assay the expression levels of *CXCL10* and *CXCL11*. Relative expression values were normalized to the *HPRT1* reference gene and non-treated cells were assigned an arbitrary value of 1. (**B**,**D**) CXCL10 and CXCL11 level was measured by ELISA. (**F**) Total RNA was isolated from WT and *PELI3*^−/−^ THP-1 cells and converted to first-strand cDNA. This was used as a template for PCR amplifying genes as indicated. Products were resolved on 1.5% (*w/v*) agarose gel electrophoresis. All results presented are representative of at least three independent experiments. * *p* ≤ 0.01 (unpaired Student’s *t*-test).

Our previous studies have shown that during VSV infection, viral particles replicate in Pellino3-knockout macrophages cell line more effectively than in wild-type cells. Increased viral replication resulting in cell lysis and death was aided by impaired synthesis of type I IFN and inflammatory cytokines production as a consequence of disturbances in the ERK1/2 pathway regulation [22]. To elucidate the role of Pellino3 in IFN-I-dependent signaling pathways, we created the THP-1 cell line with PELI3 gene knockout (*PELI3*^−/−^) using the CRISPR/Cas9 technique. We confirmed full Pellino3 knockout in THP-1 cells by Western blotting (Figure 1A) and the lack of Pellino3 protein did not affect cell viability. First, we sought to investigate the ability of Pellino3 to modulate IFNβ-induced CXCL10 and 11 production. To this end, we measured IFNAR-mediated induction of *CXCL10* and *CXCL11* expression by quantitative PCR. We observed that stimulation of wild-type THP-1 (WT) with recombinant IFNβ induced the expression of *CXCL10* and *CXCL11* genes, while Pellino3 deficiency significantly reduced the stimulatory effect of IFNβ on *CXCL10* expression but not on *CXCL11* expression. In contrast, IFNγ stimulation resulted in the comparable induction of *CXCL10* and *CXCL11* genes in both WT and *PELI3*^−/−^ cells (Figure 1B,C). Next, to confirm that mRNA assessment is reflected by the protein levels, WT and *PELI3*^−/−^ THP-1 were treated with recombinant IFNβ and CXCL10 and 11 chemokines levels were measured by ELISA. The obtained results correlated with quantitative PCR data showing that CXCL10 production was significantly decreased in *PELI3*^−/−^ THP-1 when compared to WT cells (Figure 1D). In addition, we observed that Pellino3 did not significantly affect the production of CXCL11 (Figure 1E). Importantly, the treatment of IFNβ and IFNγ cells did not affect their viability. These findings suggest that in THP-1 cells, Pellino3 preferentially modulate CXCL10 level by IFNβ-induced signaling cascades. To strengthen our hypothesis, we analyzed the expression level of the gene coding members of IFNAR. *IFNAR1* and *IFNAR2* mRNA levels were comparable in both WT and *PELI3*^−/−^ THP-1, indicating that the reported differences in cytokine induction are not associated directly with receptors’ gene expression levels (Figure 1F) but are rather related to signal transduction.

**Figure 2:**
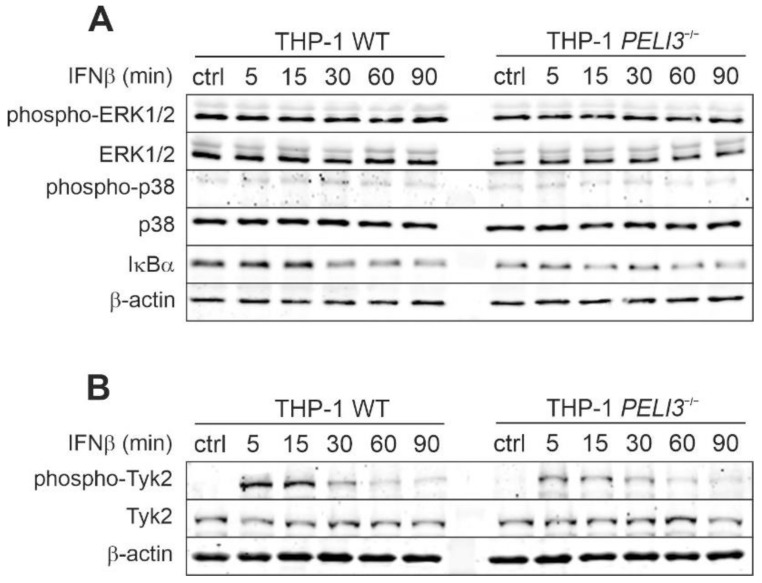
Pellino3 regulates IFNβ-induced Tyk2 phosphorylation. (**A**,**B**) Wild type (WT) and *PELI3*^−/−^ THP-1 cells were treated with IFNβ (1000 U/mL) for indicated time periods. Whole-cell lysates were subjected to SDS-PAGE followed by Western blotting. Protein detection was performed using specific antibodies and appropriate secondary antibodies conjugated to the fluorescent dye in the infrared range. Visualization was performed using the Odyssey CLx Imaging System LI-COR. The results presented are representative of at least three independent experiments.

Pellino3-regulated signaling pathways have been previously related to MAP kinase pathways [22,23]. Therefore, in the next step, we decided to examine the activation of MAPK kinases: ERK1/2 and p38. Whole cell lysates from IFNβ-stimulated WT and *PELI3*^−/−^ THP-1 cells were prepared, and the phosphorylation patterns of selected MAP kinases were analyzed by Western blotting. Interestingly, we found that neither ERK1/2 nor p38 phosphorylation was increased in response to IFNβ treatment (Figure 2A). Considering that NF-κB also drives the IFNβ-induced expression of *CXCL10* [4], we next examined the ability of Pellino3 to modulate NF-kB activation in response to IFNβ. To this end, the degradation of IκBα, the NF-κB transcription factor inhibitor was investigated. We observed only modest degradation of IκBα inhibitory protein in response to IFNβ with a similar pattern for both WT and *PELI3*^−/−^ cells.

Since Tyk2 kinase is one of the key kinases in type I IFN-dependent signaling pathways, we sought to investigate the activation of this kinase in response to IFNβ stimulation. Following ligand binding, Tyk2 is activated by phosphorylation of Tyr1054/1055 [24], which is required for the subsequent tyrosine phosphorylation of STATs [25], so in the next step, we investigated Tyk2 phosphorylation using Western blotting. Our results show that IFNβ stimulation promotes strong phosphorylation of the Tyk2 kinase in WT THP-1, while Tyk2 phosphorylation in *PELI3*^−/−^ THP-1 is impaired (Figure 2B). These findings suggest that Pellino3 can positively regulate IFNβ-induced CXCL10 production via Tyk2 activation.

### 2.2. NF-κB Positively Regulates IFNβ-Induced Expression of CXCL10 in a Pellino3-Independent Way

**Figure 3:**
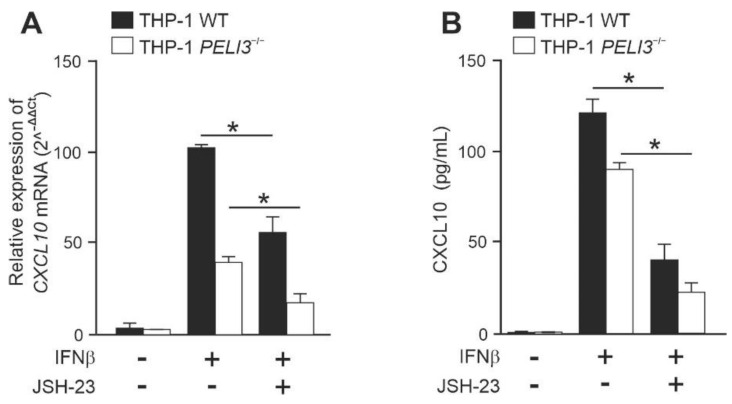
IFNβ-dependent production of CXCL10 is mediated by NF-κB, but in Pellino3 independent mechanism. Wild type (WT) and *PELI3*^−/−^ THP-1 cells were pretreated with DMSO (−/−) or JSH-23 (5 µM) for 1 h. Next, the cells were treated with IFNβ (1000 U/mL) for 4 h (**A**) or 16 h (**B**). (**A**) Thereafter, total RNA was isolated and reverse-transcribed. Quantitative real-time PCR (2^−(ΔΔCT)^) was used to assay the expression levels of CXCL10. Relative expression values were normalized to the *HPRT1* reference gene, and non-treated cells were assigned an arbitrary value of 1. (**B**) CXCL10 level was measured by ELISA. All results presented are representative of at least three independent experiments. * *p* ≤ 0.01 (unpaired Student’s *t*-test).

Given that previous experiments showed slight degradation of IκBα in response to IFNβ (Figure 2A), we investigated the role of NF-κB in Pellino3-dependent *CXCL10* expression. To this end, we used the NF-κB translocation inhibitor—JSH-23 [26]. We observed that IFNβ-induced *CXCL10* expression in WT and *PELI3*^−/−^ THP-1 was significantly suppressed in cells pretreated with inhibitor (Figure 3A). As expected, a similar effect of JSH-23 was observed on CXCL10 secretion (Figure 3B). In parallel, using Alamar Blue assay, we confirmed that these inhibitors did not change cell viability. The relative ratio of the suppression was the same in WT and *PELI3*^−/−^ cells, suggesting that NF-κB positively regulates IFNβ-induced *CXCL10* gene expression through the Pellino3-independent mechanism.

### 2.3. IFNβ-Dependent Activation of CXCL10 Requires IRF9 and STAT1/STAT2 Activation

**Figure 4:**
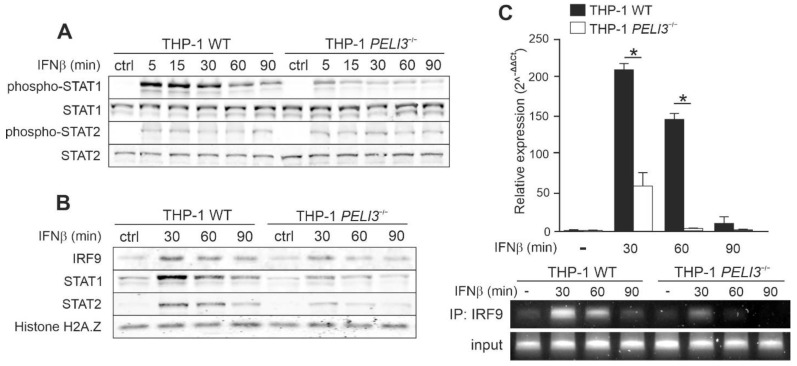
IFNβ/Pellino3-dependent activation of *CXCL10* is regulated by IRF9. (**A**–**C**) Wild type (WT) and *PELI3*^−/−^ THP-1 cells were treated with IFNβ (1000 U/mL) for indicated time periods. Whole-cell lysates (**A**) or nuclear fraction (**B**) were subjected to SDS-PAGE followed by Western blotting. Protein detection was performed using specific antibodies and appropriate secondary antibodies conjugated to the fluorescent dye in the infrared range. Visualization was performed using the Odyssey CLx Imaging System LI-COR. The results presented are representative of at least three independent experiments. (**C**) Cells were fixed with formaldehyde, followed by nuclei isolation and sonication. Sonicated nuclear lysates were immunoprecipitated with an anti-IRF9 or rabbit IgG control antibody. Input DNA (prior to immunoprecipitation) and immunoprecipitated chromatin were analyzed by quantitative real-time PCR (2^–(ΔΔCT)^) (top) and 35 cycles of standard PCR (bottom) with primers designed to amplify an ISRE binding site in the human *CXCL10* gene promoter region. Products of standard PCR were resolved on 1.5% (*w/v*) agarose gel electrophoresis. All results presented are representative of at least three independent experiments. * *p* ≤ 0.01 (unpaired Student’s *t*-test).

In canonical type I IFN-dependent pathway, STAT1 forms ISGF3 complex together with STAT2 and IRF9, which drives the expression of multiple ISGs. To investigate the ability of Pellino3 to modulate *CXCL10* induction by IFNβ at the transcriptional level, we sought to analyze the activation of the transcription factors STAT1 and STAT2. WT and *PELI3*^−/−^ THP-1 were stimulated with the recombinant human IFNβ followed by immunoblot analysis using anti-phosphorylated STAT1 and STAT2 antibodies. Only STAT1 phosphorylation was significantly suppressed in *PELI3*^−/−^ THP-1, whereas IFNβ-induced phosphorylation of STAT2 was similar in both WT and *PELI3*^−/−^ THP-1 (Figure 4A).

To reveal the mechanism of impaired *CXCL10* expression in Pellino3 deficient cells, we attempted to analyze the nuclear translocation of STAT1/STAT2/IRF9 complex in response to IFNAR activation. IFNβ promoted strong nuclear translocation of IRF9 and STAT1 (Figure 4B). Interestingly, despite the lack of differences in STAT2 phosphorylation, we observed a stronger STAT2 nuclear translocation in WT cells. We hypothesized that the formed STAT1/STAT2/IRF9 complex might initiate *CXCL10* transcription by specific binding to the ISRE element present at the *CXCL10* promoter. To confirm that hypothesis, binding of IRF9 to the *CXCL10* promoter was assayed in vivo by chromatin immunoprecipitation [27]. We observed that IFNβ promoted strong binding of IRF9 to the *CXCL10* promoter in WT compared to *PELI3*^−/−^ THP-1 (Figure 4C).

### 2.4. Knockout of Pellino3 Decreases IFNβ-Induced Expression and Production of CXCL10 in Murine Macrophage Cell Line, BMDM

**Figure 5:**
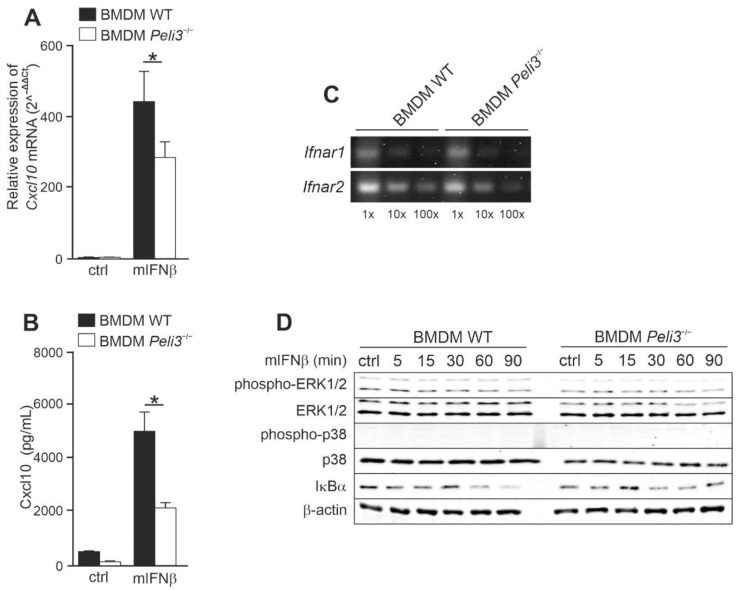
Pellino3 regulates IFNβ-induced Cxcl10 production in murine macrophages cell line, BMDM. Wild type (WT) and *Peli3*^−/−^ BMDM cells were treated with mIFNβ (mouse IFNβ) (50 ng/mL) for 4 h (**A**) or 16 h (**B**). (**A**) Thereafter, total RNA was isolated and reverse-transcribed. Quantitative real-time PCR (2^–(ΔΔCT)^) was used to assay the expression levels of *Cxcl10*. Relative expression values were normalized to the *Hprt1* reference gene and non-treated cells were assigned an arbitrary value of 1. (**B**) Cxcl10 level was measured by ELISA. (**C**) Total RNA was isolated from WT and *Peli3*^−/−^ BMDM cells and converted to first-strand cDNA. This was used as a template for PCR amplifying *Ifnar1* and *Ifnar2*. Products were resolved on 1.5% (*w/v*) agarose gel electrophoresis. (**D**) Whole cell lysates were subjected to SDS-PAGE followed by Western blotting. Protein detection was performed using specific antibodies and appropriate secondary antibodies conjugated to the fluorescent dye in the infrared range. Visualization was performed using the Odyssey CLx Imaging System LI-COR. The results presented are representative of at least three independent experiments. All results presented are representative of at least three independent experiments. * *p* ≤ 0.01 (unpaired Student’s *t*-test).

Pellino3-dependent regulation of the IFNβ-induced Cxcl10 production was also investigated in a murine system to exclude species-dependent differences in Pellino3 functionality in the context of IFNβ signaling. We used macrophages cell line derived from the bone marrow of Pellino3-deficient mice (*Peli3*^−/−^ BMDM) and WT mice (WT BMDM) [28] as a model in the following experiments. Similar to *PELI3*^−/−^ THP-1 cell line, we did not observe any significant difference in the viability of the Pellino3 deficient BMDM compared to the WT cells. Additionally, mIFNβ treatment did not affect viability of both BMDM cell lines. Cells were stimulated with mIFNβ, and the Cxcl10 expression was analyzed by qPCR. We found that in *Peli3*^−/−^ BMDM treated with mIFNβ, the expression of *Cxcl10* genes was reduced compared to WT cells (Figure 5A). As expected, the amount of Cxcl10 protein measured by ELISA was significantly lower in *Peli3*^−/−^ BMDM (Figure 5B). Next, to exclude the influence of Pellino3 knockout on *Ifnar1* and *Ifnar2* mRNA level, we analyzed the expression level of these genes. As shown in Figure 5C, neither *Ifnar1* nor *Ifnar2* expression was significantly changed in *Peli3*^−/−^ cells compared to WT cells. These results suggest that the observed impaired cytokine production by *Peli3*^−/−^ BMDM was only due to the lack of Pellino3 expression. Similar to THP-1, a modest degradation of IκBα in response to mIFNβ was observed in both WT and *Peli3*^−/−^ BMDM (Figure 5D).

**Figure 6:**
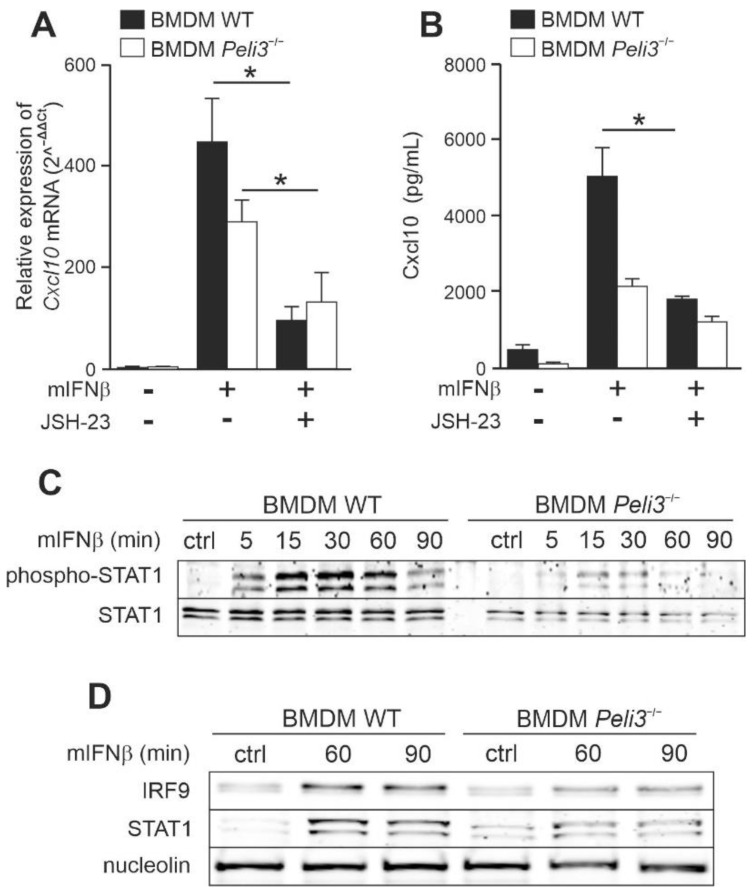
Pellino3 promotes mIFNβ-induced production Cxcl10 via IRF9-dependent pathway in BMDM cell line. Wild type (WT) and *Peli3*^−/−^ BMDM cells were pretreated with DMSO (−/−) or JSH-23 (5 µM) (**A**,**B**) for 1 hr. Next, the cells were treated with mIFNβ (mouse IFNβ) (50 ng/mL) for 4 h (**A**) or 16 h (**B**). (**A**) Thereafter, total RNA was isolated and reverse-transcribed. Quantitative real-time PCR (2^–(ΔΔCT)^) was used to assay the expression levels of *Cxcl10*. Relative expression values were normalized to the *Hprt1* reference gene and non-treated cells were assigned an arbitrary value of 1. (**B**) Cxcl10 level was measured by ELISA. (**C**,**D**) Wild type (WT) and *Peli3*^−/−^ BMDM cells were treated with mIFNβ (50 ng/mL) for indicated time periods. Whole-cell lysates (**C**) or nuclear fraction (**D**) were subjected to SDS-PAGE followed by Western blotting. Protein detection was performed using specific antibodies and appropriate secondary antibodies conjugated to the fluorescent dye in the infrared range. Visualization was performed using the Odyssey CLx Imaging System LI-COR. The results presented are representative of at least three independent experiments. All results presented are representative of at least three independent experiments. * *p* ≤ 0.01 (unpaired Student’s *t*-test).

Next, we examined the potential of NF-κB to initiate expression of the *Cxcl10* gene upon activation of mIFNβ in WT and *Peli3*^−/−^ BMDM. We again inhibited NF-κB translocation of IκBα using JSH-23. As expected, a strong suppression of mIFNβ-induced *Cxlc10* expression in both cells, pretreated with JSH-23, was observed (Figure 6A). Furthermore, secretion of the Cxcl10 was also reduced by this inhibitor (Figure 6B). Similar to THP-1, inhibitor did not influence cell viability and the relative suppression ratio was the same in WT and *Peli3*^−/−^ BMDM, suggesting that the NF-κB positively regulates mIFNβ-induced *Cxcl10* gene expression through the Pellino3-independent mechanism.

Based on the results obtained from human cell line, THP-1, we sought to determine if transcriptional factors STAT1 and IRF9 are involved in mIFNβ and Pellino3-dependent *Cxcl10* expression in BMDM cells. As shown in Figure 6C,D, after mIFNβ treatment, STAT1 was more efficiently phosphorylated in WT compared to *Peli3*^−/−^ BMDM, and the level of both STAT1 and IRF9 translocated into the nucleus was significantly abolished in the absence of Pellino3 protein.

These findings clearly indicate that the STATs-IRF9 complex activation is triggered by IFNβ and regulated by Pellino3 ligase in monocytes and macrophages cell lines.

## 3. Discussion

IFNβ, a cytokine belonging to type I IFN, modulates many cellular processes. Its presence may results in arrested viral infection, inhibited cell proliferation or modulated cell differentiation [29]. IFNβ was found to block cancer progression by limiting the recruitment of pro-angiogenic neutrophils into tumors [30]. Furthermore, IFNβ accelerates the inflammatory response of monocytes by attracting them to the sites of chronic inflammation [31]. Enhanced expression of IFNβ in DNase II-deficient embryonic macrophages has also been shown to enhance the accumulation of large amounts of DNA from apoptotic cells, thereby reducing red blood cell differentiation and leading to severe anemia [32]. IFNβ secretion is usually related to innate immune processes activated during bacterial or viral infection accompanied by the binding of a pathogen-derived ligand to PPRs (pathogen recognize receptors) such as RIG-I-like receptors (RLR) or Toll-like receptors (TLR) [33]. Consequently, the signaling cascade leading to the production and secretion of IFNβ (or other types I IFNs) is triggered [34]. The secretion of this cytokine leads to the activation of the IFNAR1/2 receptor and, consequently, to the activation of downstream signaling cascades [2]. Despite numerous studies, the signaling cascades triggered after IFNβ recognition by INFAR1/2 are not fully characterized and remain of interest to many research groups. The detailed understanding of those processes is vital since the unique properties of type I IFN (including IFNβ) speak for the application of these cytokines in antiviral, anti-cancer [35], and multiple sclerosis therapies [36]. Thus, elucidation of the new mechanisms of IFNβ regulation might lead to a better understanding of type I IFN potential in the future development of new therapies based on this cytokine.

Pellino3 ubiquitin ligase is an important regulator of the secretion of type I IFN during the innate immune response. It is known that Pellino3 negatively regulates IFNβ production in response to the activation of TLR3 and TLR4 [20,21]. Moreover, our recently reported results showed that Pelino3 could function as a positive regulator of the Cxcl10 protein production in the VSV-induced RIG-I-dependent signaling pathway [22]. Considering the already established view on the role of Pellino3 ubiquitin ligase in the regulation of type I IFN secretion, we asked whether Pellino3 could also affect signaling pathways activated by IFNβ.

In this study, we indicate that Pellino3 ligase can modify the INFAR-dependent signaling pathways upon IFNβ stimulation. We show for the first time that Pellino3 deficient monocytes and macrophages cell lines (THP-1 and BMDM, respectively) are unable to fully induce CXCL10 production in response to IFNβ.

We initially decided to examine the profile of secreted chemokines in response to IFNβ in WT and Pellino3-deficient THP-1 cell line that served as a model of human monocytes. The THP-1 cell line with *PELI3* gene knockdown (*PELI3*^−/−^) used for this research was generated with the use of the CRISPR/Cas9 technique. It is known that IFNβ promotes the expression of *CXCL10* [13] and *CXCL11* [7]. In response to IFNβ stimulation, we observed a significantly lower level of the *CXCL10* mRNA in WT cells compared to *PELI3*^−/−^, which correlated with the attenuated secretion of this cytokine. Interestingly, CXCL11 expression and secretion in *PELI3*^−/−^ cells reached the same level as in the WT cell. Additionally, the lack of Pellino3 did not change *IFNAR1* and *IFNAR2* expression, suggesting that Pellino3 does not directly affect IFNβ receptors but rather plays a role in the modulation of their signaling pathways. These data indicate that Pellino3 regulates the production of CXCL10 but does not affect the secretion of CXCL11 INFβ-induced chemokines.

To date, Pellino3 has been reported to be involved in the regulation of p38 MAP kinases [23]. Furthermore, in our previous research, we have shown that Pellino3 promotes ERK1/2 phosphorylation and IFNβ expression [22]. However, this was not reflected in our results obtained with IFNβ-treated THP-1 cells because we did not observe any activation of MAP kinases. Interestingly, we demonstrated that Pellino3 is involved in the phosphorylation of Tyk2 kinases after IFNAR1/2 activation. Since the regulation of Tyk2 kinases by ubiquitin ligases has not been described so far, our results report this mechanism for the first time.

Our study also shows that IκBα is slightly degraded in THP-1 after IFNβ stimulation, which is in line with the study of Yang et al. [4] in which IFNβ-induced expression of *CXCL10* is dependent on NF-κB. However, the inhibition of nuclear translocation of NF-κB with selective inhibitor JSH-23 resulted in the reduced IFNβ-induced expression and production of the CXCL10 in both: WT and *PELI3*^−/−^ cells. The similar inhibition ratios of the *CXCL10* expression in WT and *PELI3*^−/−^ THP-1 indicates that the process of NF-κB activation by IFNβ in monocytes cell line is independent of Pellino3 ligase. This finding suggests that IFNβ can promote the production of CXCL10 via two signaling pathways, one of which is regulated by Pellino3 and the other, independent of Pellino3, is associated with NF-κB pathways.

It is known that interferons are capable to switch macrophages from the resting-state to the activated state characterized by increased IFN-induced genes (ISGs) expression [37]. Basal expression of many ISGs is controlled by STAT2/IRF9 complexes, whose formation does not require the IFNAR1/2 receptor activation. However, type I IFN promotes creating a complete ISGF3 complex including STAT1, STAT2, and IRF9 [37,38], which is the canonical signal transduction pathway for INFβ. It has been shown that various STATs co-precipitate with IRF proteins upon IFNAR activation. In some cell types, IFN induces an immune response by activating STAT3, 4, 5, or 6 [5,34]. We have shown that IFNβ-induced phosphorylation and activation of STAT1 is suppressed in Pellino3-deficient THP-1. Importantly, this finding correlates with the ability of STAT1 to form complexes that translocate to the nucleus. We have shown that the IRF9 translocation in response to IFNβ is also positively regulated by Pellino3. Interestingly, we did not observe the effect of Pellino3 on STAT2 phosphorylation, but its translocation to the nucleus is clearly abrogated in *PELI3*^−/−^ cells. The translocation of STAT1, STAT2, and IRF9 into the nucleus in response to IFNβ indicate that the observed expression of *CXCL10* is dependent on the complete ISG3 complex. The crucial role of Pellino3 in the regulation of *CXCL10* expression via ISG3 complex was confirmed by in vivo binding of IRF9 to the *CXCL10* promoter. We demonstrated that transcription factor IRF9 binds to the regulatory element ISRE in the *CXCL10* promoter to a much lower potency in Pellino3-deficient THP-1 than in WT cells upon IFNβ treatment. These data strongly indicate that Pellino3 is involved in the regulation of IFNβ-induced production of CXCL10 mediated by STAT1/2/IRF9 complex.

We also found a similar mechanism of Cxcl10 activation in a macrophages cell line derived from the bone marrow of mice (BMDM). Our study on *Peli3*^−/−^ BMDM showed that mIFNβ treatment leads to the IFNAR-Pellino3-STAT1-IRF9-dependent secretion of Cxcl10 chemokine and allowed to exclude the possibility of species-dependent differences in Pellino3 functionality.

Although our research has focused on the role of STAT1/IRF9 and NF-κB in the activation of the *CXCL10* expression induced by IFNβ, it is clear that various transcription factors are involved in controlling the transcription of this gene. Previous studies have shown that IFNγ or dsRNA induce maximal *CXCL10* expression only when the *CXCL10* promoter sequence contains an ISRE site and two κB sites [18,39]. In turn, the activity of the *CXCL10* promoter in response to HRV-16 was reduced by ~50% following removal of the ISRE and STAT sites [14]. Therefore it has been shown that one stimuli may promote the binding of one or more transcription factors to appropriate sites in the *CXCL10* promoter sequence such as C/EBP, AP-1, ISRE, STAT, and κB sites [14,19]. It is noteworthy that our research also shows different ways to activate *CXCL10* transcription in response to IFNβ. Interestingly, we observed that simultaneous lack of Pellino3 ligase and blocked translocation of NF-κB by the JSH-23 inhibitor did not completely inhibit the IFNβ-induced expression of *CXCL10* in THP-1 and BMDM cell line. It can be supposed that other promoter clusters are also involved into the investigated mechanism. This demonstrates the complexity of the response mechanisms to type I interferons.

**Figure 7:**
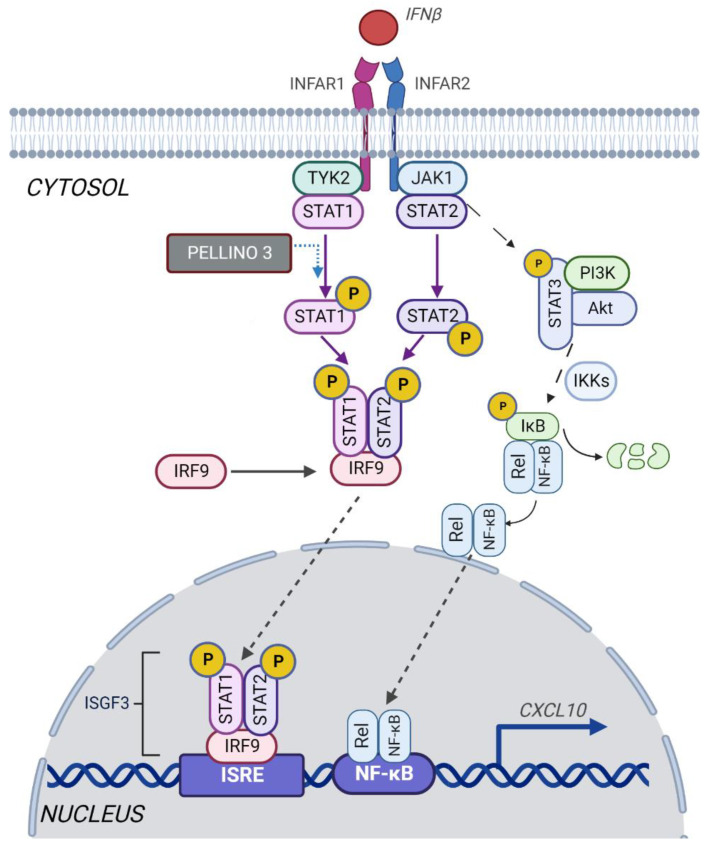
Pellino3 regulates IFNβ-induced STAT1/STAT2/IRF9- dependent activation of *CXCL10*. Figure created with biorender.com (accessed on 20 September–20 October 2022).

In conclusion, our results indicate that in BMDM and THP-1, after IFNβ treatment, expression of *CXCL10* is promoted by two independent signaling pathways. Both of them are necessary for the full activation of *CXCL10* gene transcription (Figure 7). Pellino3-independent pathway in which IFNβ induces NF-κB-mediated expression of the *CXCL10* is already well established. However, we propose a novel mechanism for the Pellino3-mediated positive regulation of IFN-induced expression of *CXCL10*. Activation of IFNAR by IFNβ results in phosphorylation of Tyk2 and the formation of STAT1/STAT2/IRF9 complex driven by STAT1 phosphorylation, which then undergoes translocation into the nucleus and promotes transcription of the *CXCL10* gene. Our study does not indicate the exact mechanism by which Pellino3 regulates IFNβ-induced *CXCL10* expression in murine macrophages and human monocytes cell lines. However, the identification of Pellino3 as a critical, positive regulator of the IFNβ-dependent CXCL10 induction is an important discovery that provides insight into the molecular mechanisms of the antiviral innate immune response induced by macrophages. In addition, our data contribute to a better understanding of the immunoregulatory function of interferons. Our results may positively contribute to the future improvement of the safety and efficacy of IFNβ-based therapy.

## 4. Materials and Methods

Cell culture and reagents—human leukemia monocytic cell line THP-1 (WT) were purchased from the European Collection of Authenticated Cell Cultures. THP-1 *PELI3*^−/−^ cells were generated using the CRISPR/Cas9 method. Immortalized BMDM cell lines from wild-type (WT), and *Peli3*^−/−^ mice were gifts from Professor Paul N. Moynagh (National University of Ireland, Maynooth, Ireland). These cell lines were generated by infecting primary bone marrow-derived macrophages cells isolated from mice with the J2 recombinant retrovirus described previously [28]. THP-1 cell lines were grown in RPMI with GlutaMAX (Gibco, Gaithersburg, MD, USA) supplemented with 10% inactivated fetal bovine serum (Sigma, St. Louis, MO, USA) and 100 µg/mL Normocin (Invivogen, San Diego, CA, USA). BMDM cell lines were grown in DMEM with GlutaMAX (Gibco) supplemented with 10% inactivated fetal bovine serum (Sigma) and 100 µg/mL Normocin (Invivogen). Cells were maintained at 37 °C in a humidified atmosphere of 5% CO_2_. Human IFNβ was purchased from Thermo Fisher Scientific. Human IFNγ and mouse IFNβ were purchased from R&D Systems (Minneapolis, MN, USA). Cell inhibitor: JSH-23 was purchased from Selleckchem (Houston, TX, USA).

Pellino3-deficient THP-1 cell line—Pellino3 knockout in THP-1 cells was generated using Guide-it CRISPR/Cas9 Gesicle Production System (Takara Bio, Kusatsu, Shiga, Jappan) according to the manufacturer’s instruction. The efficiency of genomic DNA cleavage by sgRNA/Cas9 complexes was analyzed using Guide-it Complete sgRNA Screening System (Takara Bio) according to the manufacturer’s instruction. Sequence sgRNA: 5′-GATGAGTTCACCATACTTGA-3′ was chosen as the most efficient. Pellino3 knockout was confirmed using Western blotting. Protein detection was performed using rabbit anti-human PELI3 antibody (BioRad, Hercules, CA, USA) and appropriate secondary antibodies conjugated to the fluorescent dye in the infrared range (IRDye 800CW Goat anti-Rabbit IgG (H + L) antibody, LI-COR, Lincoln, NE, USA). Visualization was performed using the Odyssey CLx Imaging System LI-COR.

First-strand cDNA synthesis—Cells were seeded in density 1 × 10^6^ cells/mL and grown for 24 h. Cells were stimulated with interferons in the following concentrations: human IFNβ—1000 U/mL, human IFNγ—15 ng/mL, and mouse IFNβ—50 ng/mL for 4 h. Cultures were incubated at 37 °C in a humidified atmosphere of 5% CO_2_. If the experiment required inhibitor administration, it was added one hour before IFNβ treatment at a final concentration of 5 µM. Total RNA was isolated using TRI Reagent (Sigma) according to the manufacturer’s protocol. Isolated RNA (1 µg) was incubated with DNase I (Thermo Fisher Scientific, Waltham, MA, USA) at 37 °C for 30 min. Then, DNase I was inactivated by the addition of 50 mM EDTA and incubation at 60 °C for 10 min. Thereafter, cDNA was synthesized using iScript reverse transcription supermix for RT-PCR (Bio-Rad), accordingly to the manufacturer’s instructions. Reactions were incubated at 25 °C for 5 min, followed by 46 °C for 20 min, and heated to 95 °C for 1 min.

PCR and quantitative real-time PCR—Total cDNA (10 ng) was used for qPCR with CFX Connect qPCR system (Bio-Rad) and iTaq Universal SYBR Green Supermix (Bio-Rad). For each mRNA quantification, the housekeeping gene hypoxanthine phosphoribosyltransferase 1 (*HPRT1* or *Hprt1*) was applied as a reference point. Real-time PCR data were analyzed using the 2^−(ΔΔCT)^ method. Conventional PCR was performed using DNA REDTaq polymerase (Sigma) with 70 ng of total cDNA according to the manufacturer’s protocol. PCR products were resolved by 1.5% (*w/v*) agarose gel electrophoresis and then analyzed using a Gel Doc (Bio-Rad).

For the amplification of the specific genes the following primers were used: *CXCL10,* forward: GGAGATGAGCTAGGATAGGATAGAGGG, reverse: TGCCCATTTTCCCAGGACCG; *CXCL11,* forward: CTACAGTTGTTCAAGGCTTC, reverse: CACTTTCACTGCTTTTACCC; *HPRT1*, forward: AGCTTGCTGGTGAAAAGGAC, reverse: TTATAGTCAAGGGCATATCC; *IFNAR1,* forward: AGTTGAAAATGAACTACCTCC, reverse: ACTTGAAAGGTCATGTTTGC; *IFNAR2*, forward: CATGTCTTTTGAACCACCAG, reverse: CTTAACAATCCCTCTGACTG; *Cxcl10*, forward: GCCATGGTCCTGAGACAAA, reverse: AGCTTACAGTACAGAGCTAGGA, *Hprt1,* forward: GCTTGCTGGTGAAAAGGACCTCTCTCGAAG, reverse: CCCTGAAGTACTCATTATAGTCAAGGGCAT; *Ifnar1*, forward: TGTTTATGTCAACTGTCAGG, reverse: TCCTTCTCCATGCTTATCTTAG; *Ifnar2*, forward: GTACACAGTCATGAGCAAAG, reverse: TCCAACCACTTATCTGTCAC.

Chromatin immunoprecipitation assay—WT and *PELI3*^−/−^ THP-1 cells were seeded at density 1 × 10^6^ cells/mL in 6-well plates and grown for 24 h to confluency. Cells were stimulated with 1000 U/mL IFNβ for 30, 60, and 90 min. Cultures were incubated at 37 °C in a humidified atmosphere of 5% CO_2_. Next, cells were fixed in formaldehyde, followed by nuclei isolation and sonication. Sonicated nuclear lysates were immunoprecipitated with an anti-human IRF9 or rabbit IgG control antibody, as previously described [27]. Input DNA (prior to immunoprecipitation) and immunoprecipitated chromatin were analyzed by quantitative real-time PCR (2^–(ΔΔCT)^) and standard PCR using specific primers designed to amplify an ISRE binding site in the human CXCL10 gene promoter region. The primers were as follows: forward: 5′-AGAAACAGTTCATGTTTTGGAAAGT-3′ and reverse: 5′-AAGTCCCATGTTGCAGACTCG-3′. Standard PCR products were resolved by 1.5% (*w/v*) agarose gel electrophoresis and then analyzed using a Gel Doc (BioRad).

ELISA—Cells were seeded in density 1 × 10^6^ cells/mL and grown for 24 h. Then, cultures were stimulated with interferons in the following concentrations: human IFNβ—1000 U/mL; human IFN γ—15 ng/mL, and mouse IFNβ—50 ng/mL, for 16 h. If the experiment required inhibitor administration, it was added one hour before IFN treatment at a final concentration of 0.5 µM. Cultures were incubated at 37 °C in a humidified atmosphere of 5% CO_2_. CXCL10, CXCL11, and Cxcl10 concentration was measured in the harvested medium from overstimulated cells by DuoSet ELISA (R&D System) according to the manufacturer’s instruction. ELISA tests were performed by the automated system E-LizaMat X-2 (DRG International, Springfield, NJ, USA).

Western blotting—Cells were seeded (1 × 10^6^ cells/mL) and grown for 24 h. Then, cultures were stimulated with interferons in the following concentrations: human IFNβ—1000 U/mL; mouse IFNβ—50 ng/mL for 5, 15, 30, 60 and 90 min. Whole-cell lysates: Cells were washed with ice-cold PBS and lysed in RIPA buffer (30mM HEPES, pH 7.4, 150 mM NaCl, 1% Nonidet P-40, 0.5% sodium deoxycholate, 0.1% sodium dodecyl sulfate, 5 mM EDTA) supplemented with protease inhibitors Complete Mini Tablets (Roche, Basel, Switzerland) and phosphatase inhibitors PhosSTOP (Roche) on ice for 30 min. Nuclear fraction: Cells were washed with ice-cold PBS and disintegrated in ice-cold buffer A (10 mM HEPES pH 7.9, 10 mM KCl, 0.1 mM EDTA, 0.1 mM EGTA, 1 mM DTT, 1 mM PMSF, and 0.1mM sodium orthovanadate, 0.1% NP-40) on ice for 15 min. After centrifugation at 12,000 g for 1 min at 4 °C, the supernatants were removed, and the nuclear pellets were resuspended in 3× the packed nuclear volume of ice-cold high-salt buffer B (20 mM HEPES pH 7.9, 10 mM KCl, 1 mM EDTA, 1 mM EGTA, 420 mM NaCl, 20% glycerol, 1 mM DTT, 1 mM PMSF). The samples were gently vortexed at 4 °C for 30 min, centrifuged at 12,000 g for 10 min at 4 °C, and the supernatants (the nuclear fraction) were saved. All cell lysates were subjected to SDS-PAGE followed by Western blot analysis with anti-GAPDH, anti-phospho-ERK1/2, anti-ERK1/2, anti-phospho-p38, anti-p38, anti-IκBα, anti-phospho-STAT1, anti-STAT1, anti-phospho-STAT2, and anti-STAT2 antibodies, anti-IRF9, anti-Histone H2A.Z (Cell Signaling, Danvers, MA, USA), anti-β-actin (Sigma), anti-nucleolin (Santa Cruz Biotechnology, Dallas, TX, USA), and secondary antibodies: IRDye 800CW Goat anti-Rabbit IgG (H + L), IRDye 800CW Goat anti-Mouse IgG (H + L) (LI-COR). Imaging was performed using ODYSSEY CLx Infrared Imaging System (LI-COR).

Data analysis—Statistical analysis was carried out using the unpaired Student’s *t*-test using GraphPad Prism 7.04. *p* values of less than or equal to 0.01 were considered to indicate a statistically significant difference (* *p* ≤ 0.01).

## 5. Conclusions

In this study, we indicate for the first time that Pellino3 plays an essential role in IFNAR1/2-dependent production of the CXCL10 chemokine in IFNβ-stimulated monocytes and macrophages cell lines (THP-1 and BMDM, respectively). Our results show that IFNβ stimulation leads to Pellino3-dependent phosphorylation of TYK2. Simultaneously, we observed Pellino3-dependent STAT1 phosphorylation and translocation of the STAT1/STAT2/IRF9 complex to the nucleus, which is essential for the expression and synthesis of CXCL10.
